# Acoustic localization of terrestrial wildlife: Current practices and future opportunities

**DOI:** 10.1002/ece3.6216

**Published:** 2020-06-13

**Authors:** Tessa A. Rhinehart, Lauren M. Chronister, Trieste Devlin, Justin Kitzes

**Affiliations:** ^1^ Department of Biological Sciences University of Pittsburgh Pittsburgh PA USA

**Keywords:** acoustic localization system, autonomous recording units, bioacoustics, conservation, microphone array, wildlife monitoring

## Abstract

Autonomous acoustic recorders are an increasingly popular method for low‐disturbance, large‐scale monitoring of sound‐producing animals, such as birds, anurans, bats, and other mammals. A specialized use of autonomous recording units (ARUs) is acoustic localization, in which a vocalizing animal is located spatially, usually by quantifying the time delay of arrival of its sound at an array of time‐synchronized microphones. To describe trends in the literature, identify considerations for field biologists who wish to use these systems, and suggest advancements that will improve the field of acoustic localization, we comprehensively review published applications of wildlife localization in terrestrial environments. We describe the wide variety of methods used to complete the five steps of acoustic localization: (1) define the research question, (2) obtain or build a time‐synchronizing microphone array, (3) deploy the array to record sounds in the field, (4) process recordings captured in the field, and (5) determine animal location using position estimation algorithms. We find eight general purposes in ecology and animal behavior for localization systems: assessing individual animals' positions or movements, localizing multiple individuals simultaneously to study their interactions, determining animals' individual identities, quantifying sound amplitude or directionality, selecting subsets of sounds for further acoustic analysis, calculating species abundance, inferring territory boundaries or habitat use, and separating animal sounds from background noise to improve species classification. We find that the labor‐intensive steps of processing recordings and estimating animal positions have not yet been automated. In the near future, we expect that increased availability of recording hardware, development of automated and open‐source localization software, and improvement of automated sound classification algorithms will broaden the use of acoustic localization. With these three advances, ecologists will be better able to embrace acoustic localization, enabling low‐disturbance, large‐scale collection of animal position data.

## Graphical abstract

Acoustic localization can be used to locate a vocalizing animal spatially using an array of time‐synchronized microphones. We comprehensively review applications of acoustic localization for terrestrial wildlife. We describe trends in the literature, identify considerations for field biologists who wish to use these systems, and suggest advancements that will improve the field of acoustic localization.

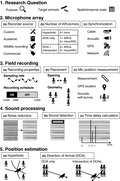

## 1 INTRODUCTION

Autonomous sensing methods are transforming data collection in ecology and conservation biology. Indirect, technology‐mediated observation is increasingly complementing, or supplanting, human observers in the field. These methods include autonomous sensors, such as camera traps, acoustic recorders, and satellite imagery, and may involve automated review, such as machine learning models that identify the species present in large data streams (Peters et al., 2014). Automated methods have the potential to survey more locations and remain in the field for longer periods than human observers, radically increasing the spatiotemporal coverage of available biodiversity data (Kitzes & Schricker, 2019).

Of these new automated approaches, autonomous recording units (ARUs) show particular promise for surveying sound‐producing taxa, including terrestrial animals such as birds, bats, amphibians, and insects, and aquatic animals such as cetaceans. Many species in these groups are important model systems for biologists and are of specific conservation concern. ARUs are more cost‐effective for large‐scale, high‐resolution wildlife surveys than human observers (Darras et al., 2019) and in many cases their performance meets or exceeds that of human surveyors (Darras et al., 2019; Simons, Alldredge, Pollock, & Wettroth, 2007). Use of these methods at large scales is becoming even more practical, thanks to advances in inexpensive recording technology, such as the AudioMoth open‐source ARU (Hill et al., 2017) and Raspberry Pi‐based ARUs (Beason, Riesch, & Koricheva, 2019; Segura‐Garcia, Felici‐Castell, Perez‐Solano, Cobos, & Navarro, 2015; Whytock & Christie, 2017). The recordings generated by ARUs can be kept as a long‐lasting historical record. These data can be reanalyzed in the future to apply updated analysis techniques or to answer new questions.

A less common application of autonomous recording is acoustic localization, the use of multiple time‐synchronized ARUs to estimate an animal's location by quantifying the time difference of arrival (TDOA, also time delay of arrival) of its sound at each microphone. This process, also known as *acoustic multilateration*, gained popularity as a method of studying the behavior and ecology of underwater animals, which are challenging to directly observe (e.g., Spiesberger & Fristrup, 1990; Watkins & Schevill, 1972). Knowing an animal's location broadens the use of ARUs, allowing researchers to generate abundance and density estimates (e.g., Wahlberg, Tougaard, & Møhl, 2003), observe habitat usage (e.g., Wilson & Bayne, 2018), calibrate acoustic indices (e.g., Thompson, Schwager, Payne, & Turkalo, 2009), study animal behavior and communication (e.g., Collier, Blumstein, Girod, & Taylor, 2010), and track animal movements across small and large scales (e.g., Kershenbaum, Owens, & Waller, 2019). Aside from wildlife surveys, applications of localization in conservation include locating poachers by the sounds of gunshots or locating illegal logging by the sounds produced by chainsaws (e.g., Andrei, 2015; Wijers, Loveridge, Macdonald, & Markham, 2019).

There is little standardization in techniques for acoustic localization of terrestrial wildlife, and the field lacks a comprehensive review. We are aware of three general discussions of localization of terrestrial wildlife in prior literature. Blumstein et al. (2011) describe overarching requirements, goals, and applications of acoustic monitoring, with a section identifying commonly used methods of localization. This review also identifies future directions for the field of acoustic monitoring in general. A similar analysis by Huetz and Aubin (2012) describes the principles of localization methods and describes an example localization method. Lastly, Koblitz (2018) describes applications of localization to bat echolocation in particular. However, none of these three reviews attempted to comprehensively survey the literature to summarize current uses of terrestrial localization, identify best practices for its use by ecologists, and suggest future directions to advance research in the field of acoustic localization.

In this paper, we comprehensively review published applications of acoustic localization of wildlife in terrestrial environments. We do not consider aquatic localization, as several prior reviews describe techniques for localization of aquatic wildlife (Mellinger, Stafford, Moore, Dziak, & Matsumoto, 2007; Van Parijs et al., 2009), and these techniques are substantially different than those used for terrestrial wildlife. We identify the steps used to design a localization study and describe the variety of approaches for completing each step. We discuss three features of the literature, including eight purposes for localization systems, the strengths and weaknesses of the two broad methods of localization, and an overall lack of automated localization methods. We also describe considerations for field biologists who wish to implement acoustic localization systems. Finally, we suggest three priorities for future work: increased availability of inexpensive time‐synchronized recorders, development of localization software that can localize sounds in dense soundscapes, and automated classification of animal sounds.

## 2 MATERIALS AND METHODS

We conducted a review of all applications of acoustic localization using autonomous recording units in terrestrial environments. In early 2020, we searched Web of Science with the following query:

TOPIC: ((localization OR localisation OR tdoa OR doa OR beamform*) AND (acoustic OR microphone* OR aru) AND (ecolog* OR conservation OR animal* OR bird* OR bat* OR mammal* OR avian))

This search returned 827 results. We inspected the returned abstracts to identify papers in which acoustic localization was used to localize wildlife in terrestrial environments or in which an acoustic localization system for this purpose was tested. For papers that included components not conducted in the field, such as computer simulations (e.g., Chen, Ali, & Wang, 2006; Park & Kotun, 2018) or captive bats in a flight room (e.g., Surlykke, Pedersen, & Jakobsen, 2009), only field components were assessed. In addition to the papers retrieved from Web of Science, we included a set of papers of which we were already aware. We then recursively searched for literature that referenced or was referenced by the papers in our collection. Because we focused on systems for studying wildlife in natural environments, we did not include papers where animals were captive, such as birds kept for falconry (Sarradj, Fritzsche, & Geyer, 2011), domesticated animals (Du, Lao, & Teng, 2018; Silva et al., 2008), or animals localized within a laboratory setting (e.g., Clark & Mistick, 2018; Falk, Jakobsen, Surlykke, & Moss, 2014; Warren, Sangiamo, & Neunuebe, 2018). We located a total of 95 studies that met these criteria (Table S1). Common terminology used in the literature is defined in a glossary (Table 1).

**Table 1:** Glossary of key terms in bioacoustics and acoustic localization

Term	Definition
Autonomous recording unit (ARU)	Device constructed of one or several microphones that are rigidly attached to each other in one configuration (see Section “Number of ARUs and microphones”)
Amplitude	Maximum change in air pressure caused by a sound wave. Correlated with perception of a sound's loudness
Array	One or multiple time‐synchronized autonomous recording units
Classification	Process of identifying what species or individual organism produced a sound
Direction of arrival (DOA) localization	Localization of sound using far‐field assumption. One ARU estimates the direction from which sound arrived. Multiple DOA estimates can be intersected to identify a coordinate location
Directionality	Degree to which a sound is not equally loud in all directions from the source
Far‐field assumption	Assumption that sound arrives at microphones as a planar wave. Typically used when distance between microphones is much smaller than distance to source
Frequency	The number of oscillations per second of a sound, measured in Hertz (Hz). High‐frequency sounds are perceived as high‐pitch sounds; low‐frequency sounds are perceived as low‐pitch
Hyperbolic localization	Localization of sound using near‐field assumption. Determines the sound's coordinate location by plotting it on multiple hyperbolas, each generated from the time difference of arrival of a sound at a pair of microphones
Microphone	Device for converting sound into an electronic signal. Sometimes known as a *receiver* or a *sensor*
Near‐field assumption	Assumption that sound arrives at microphones as a spherical wave. Used when the distance between the microphones is the same order of magnitude as the distance between the sound source and the microphones
Sample rate	Rate at which electronic signal of a microphone is sampled to be saved to a digital audio file. Higher sample rates can capture sound produced at higher frequencies
Soundscape	Combination of all biological, geological, and anthropogenic sound present in an environment at a given time (Pijanowski, Farina, Gage, Dumyahn, & Krause, 2011)
Source separation	Separation of one or multiple target sounds from each other and from background noise present in the soundscape
Spectrogram	Visual representation of sound, displaying sound amplitude at each time and frequency interval

## 3 RESULTS

**Figure 1:**
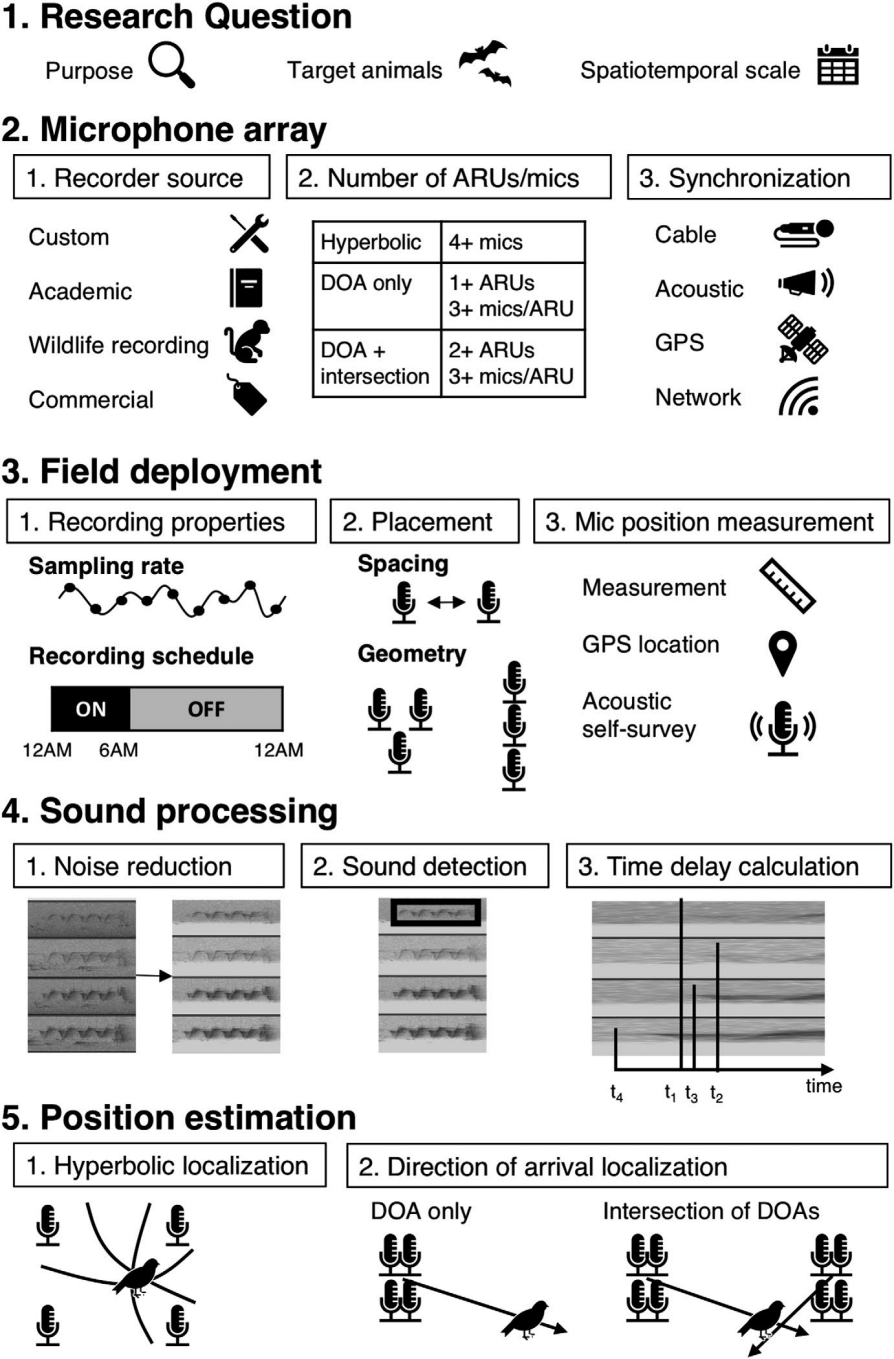
Process of acoustic localization. First, a research question is defined, including a purpose for localization, target animals to be localized, and the study's spatiotemporal scale. Second, a time‐synchronizing microphone array is obtained or built. Arrays are designed to be capable of either hyperbolic or direction‐of‐arrival (DOA) localization. Third, the microphone array is set up and deployed in the field to record ambient sound. Fourth, after the microphone array returns from the field, its recordings, represented here as spectrograms, are processed by noise reduction, sound detection, and TDOA calculation methods. Fifth, an algorithm uses the relationship between these sounds to locate the source

The acoustic localization process consists of five steps: defining a research question, obtaining or building a time‐synchronizing microphone array, deploying the array in the field to record sounds, processing the recordings captured in the field, and determining animal location using position estimation algorithms (Figure 1).

Performing an acoustic localization study requires knowledge of the properties of sound, including the speed of sound, frequency, wavelength, and amplitude. Sound is a periodic vibration of physical matter. When a sound is produced in air, air particles compress, and decompress, in waves of pressure radiating outward from the sound source. First, the speed of sound in air is the speed at which these waves of pressure travel, determined largely by the temperature, humidity, and overall pressure of the air. For instance, sound travels at a speed of about 343 meters per second at room temperature (20°C) in dry air at sea level, and about 338 meters per second in similar conditions at 10°C. In practice, the effects of humidity and air pressure are often ignored while calculating the speed of sound, as their impact on speed of sound is usually small compared to the effect of temperature variation (Spiesberger & Fristrup, 1990; Woelfel & McDonough, 2009). Therefore, speed of sound is typically calculated using an equation similar to the following (from Wilson, Battiston, Brzustowski, & Mennill, 2014):

$$\text{Speed of sound}\left(\text{m/s}\right)=331.5*\sqrt{1+\frac{\text{temperature}\left({}^{\circ }C\right)}{273.15}}$$

The above is the equation for the speed of sound in still air. If air is moving due to wind, then sound will travel faster to downwind locations and slower to upwind locations than estimated by this equation. Generally, localization is more precise when atmospheric conditions are accounted for, which may be especially important in windy conditions or in humid environments such as rainforests (Spiesberger & Fristrup, 1990). Second, the sound's frequency, measured in Hertz (Hz), is the number of waves produced per second. Fast, high‐frequency vibrations are perceived as high sounds, such as a squeak, and slow, low‐frequency vibrations are perceived as low sounds, such as thunder. Sounds like a whistle or pure tone occupy a narrow range of frequencies, whereas sounds like a clap occupy a wide range of frequencies. Third, the sound's wavelength is the distance between the waves. Wavelength can be calculated by dividing the speed of sound by the sound's frequency. For instance, a pure tone of 3,400 Hz traveling at 340 m per second has a wavelength of 0.1 m. Wavelength and frequency are inversely proportional with high‐frequency sounds having shorter wavelengths. Finally, a sound's amplitude is the maximum change in air pressure caused by a sound wave. All else being equal, the higher the amplitude of an audible sound is, the louder it is perceived to be. Many sources use “amplitude” to refer interchangeably to closely related quantities, including intensity, sound pressure level, and loudness. In air, the amplitude of a sound decreases farther from the source of the sound, a process called *attenuation*. Higher frequencies attenuate faster than lower frequencies, meaning that for two sounds produced at the same amplitude but different frequencies, the lower‐frequency sound can be heard from a greater distance than the higher‐frequency sound, although these effects are mediated by factors such as habitat and weather conditions (Goerlitz, 2018; Priyadarshani, Castro, & Marsland, 2018; Spiesberger & Fristrup, 1990).

Acoustic localization uses recordings captured by an array of time‐synchronized ARUs to estimate the position of a sound source. After an animal makes a sound, the sound's arrival at each microphone is delayed by an amount of time. This time delay is equal to the distance the sound travels from the source to the microphone, divided by the speed of sound in that environment. For instance, a sound that travels 34 m at a speed of 340 m per second will take 0.1 s to arrive at a microphone. The distance traveled from source to microphone depends on whether the sound travels straight through the air from the source to the microphone, or is reflected off of another surface, such as a tree or the ground, before being received at the microphones. Reflection increases the distance the sound travels. Terrestrial localization generally relies on receiving sounds directly from the source, although some marine applications of localization utilize indirect signals reflected off of the water's surface (Tiemann, Thode, Straley, O’Connell, & Folkert, 2006). Typically, microphones used in terrestrial localiation are omnidirectional (capable of receiving sound from all directions) and are positioned away from barriers such as tree trunks, which block the direct arrival of sounds produced behind the barrier. Reflection and reverberation due to vegetation or manmade obstacles are common causes of inaccurate position estimation. Some studies outside of the wildlife localization literature have applied measurements of the reverberation of the environment to improve position estimation accuracy (e.g., Gustafsson, Rao, & Trivedi, 2003).

Because the sound travels a different distance to reach each microphone, the sound arrives at each microphone at a slightly different time. The time difference of arrival (TDOA, sometimes abbreviated TOAD) of a sound is the difference between the sound's arrival times at two microphones. The TDOA is a function of the sound source's relative distance from each microphone, with TDOAs being larger for sounds that are much closer to one microphone than another. The TDOA between each pair of recorders is slight, on the order of tenths of a second in a typical application. To accurately capture these small differences, recorders must be synchronized within milliseconds of each other (Mennill, Battiston, Wilson, Foote, & Doucet, 2012).

**Figure 2:**
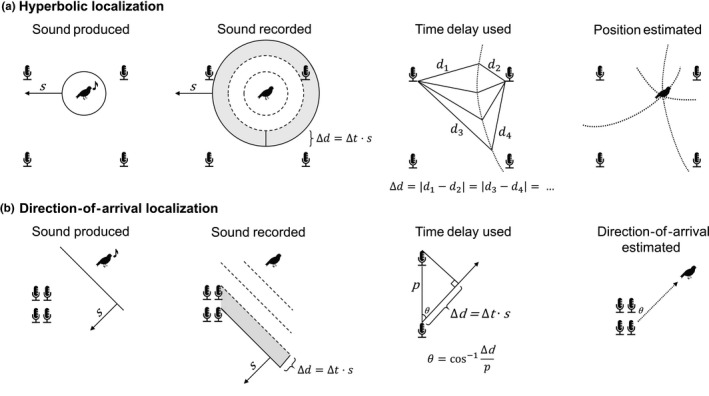
Differences between hyperbolic and direction‐of‐arrival localization in two dimensions. (a) Two‐dimensional hyperbolic localization assumes that sound arrives at each microphone as a circular front. The sound travels a slightly different distance before arriving at each microphone. The difference in distance, illustrated for two recorders, is equal to the difference in the sound's arrival time at each recorder, Δ*t*, multiplied by the speed of sound, *s*. This difference defines a hyperbola of possible source locations. The intersection of multiple hyperbolas estimates source location. (b) In the two‐dimensional case, direction‐of‐arrival localization assumes that sound arrives at the microphones as a straight front. The difference in the distance the wave travels to two recorders, Δ*d*, is illustrated. The angle of the sound's arrival is derived from the inverse cosine of Δ*d* divided by the spacing *p* between the two recorders. Each angle measurement defines a cone of potential source locations, where the cone's axis is centered on the line between the two recorders. Cones arising from multiple angle measurements are intersected to estimate the direction that the sound arrived from

Localization approaches divide into two broad categories: hyperbolic and direction of arrival (DOA; Figure 2). Both approaches can localize animals in two‐ or three‐dimensional space. The methods differ in whether the sound is assumed to be in the near field (hyperbolic) or the far field (DOA), a choice which roughly corresponds to different needs in hardware, sound processing methods, and position estimation software. Which assumption is appropriate depends on the distance between the source and any given microphone relative to the distances between the microphones themselves. When a sound is emitted, sound waves radiate from the source location in a spherical pattern. However, when a distant sound arrives at microphones that are positioned close to each other, the curved edge of the sound's arrival can be approximated as a straight line or plane. Hyperbolic algorithms make a near‐field assumption, assuming that the sound can be represented as propagating circularly (in two dimensions) or spherically (in three dimensions). This method calls for widely spaced arrays, such that the distance between the sound source and any given ARU is about the same order of magnitude as the distance between the ARUs (Koblitz, 2018). These algorithms are referred to as “hyperbolic” due to the hyperbolic solutions arising from plotting information from TDOAs on a two‐dimensional surface (Figure 2; see also Militello & Buenafuente, 2007). These methods often require the explicit calculation of TDOAs, so are referred to as “TDOA localization algorithms” in much of the wildlife localization literature. Conversely, DOA algorithms make the far‐field assumption, meaning that the sound is assumed to be far enough away that its arrival at the microphones can be approximated as a straight front (in two dimensions) or as a planar front (in three dimensions). These algorithms typically employ ARUs containing four or more closely spaced microphones, where a single ARU is only capable of finding direction of arrival, not a coordinate location. Methods which find the direction of arrival of a sound instead of its coordinate location are appropriate for distinguishing songs produced by spatially separated individuals, or separating sounds from background noise. Additionally, coordinate location of the sound source can be found by intersecting direction‐of‐arrival estimates from two or more ARUs. Other methods for acoustic localization, such as time‐of‐arrival and amplitude‐based localization (see Cobos, Antonacci, Alexandridis, Mouchtaris, & Lee, 2017; Rascon & Meza, 2017), were not used in any of the literature reviewed here. These methods are impractical for use in localizing wildlife. The time‐of‐arrival method can be used when the user knows the true time a sound was emitted (Cobos et al., 2017), a value that is unknown for wildlife applications. Amplitude‐based localization, which compares a sound's amplitude at each microphone of a multimicrophone array, suffers from inaccuracies at long distances or in field environments. However, this method could be used to determine a general bearing for the sound, such as whether it is in front of or behind the array (Rascon & Meza, 2017).

Below, we discuss each of the five major steps in the process of localization (Figure 1). For each step, we identify the necessary decisions to complete each step, as well as the options available in the literature for making these decisions.

### 3.1 Research question

The research question of a study encompasses the purpose of localization, what animals are the targets of localization, and the spatiotemporal scale of the study (Figure 1).

**Figure 3:**
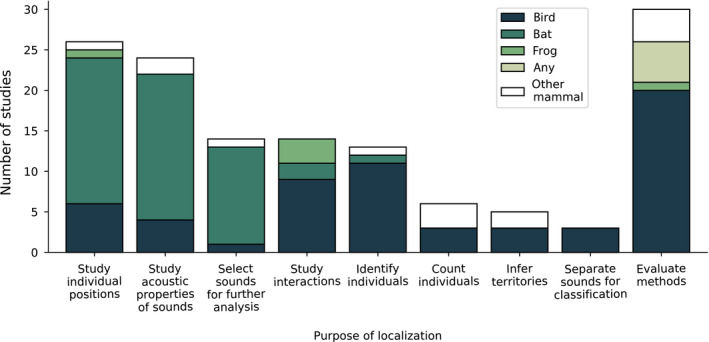
Studies organized by purpose of localization and taxon localized. Each study fell under at least one of the following categories: animal behavior, bioacoustics, population monitoring, physiology, and methods development. Taxa include birds, bats, frogs, and “other mammals,” which include elephants, marmots, orangutans, and wolves. Some studies tested methods that could be used to localize any animal

Localization was used for eight purposes in animal behavior and ecology, with 35 studies using localization for multiple purposes (Figure 3). Twenty‐six studies assessed individual animals' positions or movement, such as responses to conspecific or interspecific disturbances (e.g., Campbell & Francis, 2012; Collier, Blumstein, et al., 2010; Langemann, Peake, Tavares, & McGregor, 2000), flight speed or style in bats (e.g., Grodzinski, Spiegel, Korine, & Holderied, 2009; Ing et al., 2016; Miller & Treat, 1993), positions of displaying male birds and frogs (e.g., Grafe, 1997; Patricelli & Krakauer, 2010), and determining the position of individual predator bats when insects' auditory organs perceived these predators (Goerlitz, ter Hofstede, Zeale, Jones, & Holderied, 2010; Roeder, 1966; Schul, Matt, & Helversen, 2000). Twenty‐four studies quantified the amplitude or directionality of animal sounds, using localization to account for the animal's distance or position in relation to the microphone; this method was especially common in studies of bats (e.g., Holderied & Helversen, 2003; Jakobsen, Olsen, & Surlykke, 2015; Lewanzik & Goerlitz, 2018), but was also used to study elephants (Hedwig, DeBellis, & Wrege, 2018; Wrege, Rowland, Keen, & Shiu, 2017) and birds (Dantzker, Deane, & Bradbury, 1999; Patricelli, Dantzker, & Bradbury, 2007, 2008). Fourteen studies used localization to select subsets of sounds for further acoustic analysis, such as selecting calls from flights where bats approached the microphone array at a desired angle (e.g., Motoi, Sumiya, Fujioka, & Hiryu, 2017; Sumiya, Fujioka, Motoi, Kondo, & Hiryu, 2017). Fourteen studies localized multiple individuals simultaneously to study their behavior during interactions, such as interactions between pairs or rivals (e.g., Foote, Fitzsimmons, Mennill, & Ratcliffe, 2008; Mennill & Vehrencamp, 2008). Thirteen studies used localization to determine animals' individual identities (e.g., Krakauer et al., 2009; Lippold, Fitzsimmons, Foote, Ratcliffe, & Mennill, 2008). Six studies calculated animal abundance, either by direct calculation of number of individuals (Frommolt & Tauchert, 2014; Hedley, Huang, & Yao, 2017; Spillmann et al., Willems, van Noordwijk, Setia, & van Schaik, 2017; Wahlberg et al., 2003; Wilson & Bayne, 2018) or indirectly by calibration of acoustic indices, as described by Stevenson et al. (2015) (Thompson et al., 2009). Five studies used localization to infer territory boundaries or habitat use, including assessing animals' relationships with anthropogenic or natural habitat features (Ethier & Wilson, 2019; Hennigar, Ethier, & Wilson, 2019; Kershenbaum et al., 2019; Spillmann et al., 2017; Wilson & Bayne, 2018). Three studies separated animal sounds from background noise to improve species classification (Kojima, Sugiyama, Hoshiba, Suzuki, & Nakadai, 2017; Kojima, Sugiyama, Suzuki, Nakadai, & Taylor, 2016; Suzuki, Matsubayashi, Nakadai, & Okuno, 2016). Besides these eight explicitly biological purposes, 30 studies were tests of localization methods or included such tests as a major goal.

The research question also encompasses what animals are the targets of localization, including whether one or multiple species will be localized, and whether one or multiple individuals will be localized. The most commonly localized taxonomic groups were birds (46 papers, 48% of studies) and bats (28 papers, 29% of studies) (Figure 3). Other animals studied included frogs (5 papers), elephants (4 papers), marmots (3 papers), orangutans (2 papers), and canids (2 papers). Five papers described localization approaches that could be applied to any terrestrial animal. Of the 95 studies, 33 localized multiple species (e.g., Campbell & Francis, 2012; Kojima et al., 2017; Surlykke & Kalko, 2008). Thirty studies performed simultaneous localization of multiple individuals across a large spatial extent (e.g., Araya‐Salas, Wojczulanis‐Jakubas, Phillips, Mennill, & Wright, 2017; Campbell & Francis, 2012; Foote, Ratcliffe, Mennill, & Fitzsimmons, 2010; Lapierre, Mennill, & MacDougall‐Shackleton, 2011; Patricelli & Krakauer, 2010; Suzuki et al., 2018).

The last aspect of a research question is its spatial and temporal scale. Both the purpose of the study and the characteristics of the target sounds influence the spatial scale for each study. Studies across multiple territories used large grids of arrays to record multiple individuals simultaneously in some cases (e.g., Fitzsimmons, Foote, Ratcliffe, & Mennill, 2008a; Lapierre et al., 2011), and in others studied smaller areas individually, such as by repositioning arrays over time (e.g., Araya‐Salas et al., 2017). Bioacoustic studies tended to occur over smaller areas (e.g., Fujioka, Mantani, Hiryu, Riquimaroux, & Watanabe, 2011; Patricelli, Dantzker, et al., 2008). Loud, far‐ranging animals such as wolves and orangutans were localized on arrays covering large spatial extents (e.g., Kershenbaum et al., 2019; Papin, Pichenot, Guérold, & Germain, 2018), whereas the quickly attenuating vocalizations of bats were typically localized using arrays that surveyed smaller areas (e.g., Ratcliffe, Jakobsen, Kalko, & Surlykke, 2011). Spatial scale of arrays is discussed in greater depth in Section “Placement”. Duration of a study varies from a single recording session to assess bioacoustic traits of a species, to weeks or months of monitoring to map territories or habitat use (e.g., Spillmann et al., 2017). Population monitoring studies often drew on multiple years of data (e.g., Frommolt & Tauchert, 2014; Kershenbaum et al., 2019; Thompson et al., 2009; Wilson & Bayne, 2018).

### 3.2 Microphone array

After defining the purpose of the research, a microphone array suitable for the study must be assembled (Figure 1). An array consists of one or more time‐synchronized autonomous recording units (ARUs). Three considerations for constructing this array are the source of the recording equipment, the number of ARUs to use and microphones per ARU, and the method of synchronizing multiple ARUs.

#### 3.2.1 Recorder source

Microphone arrays can be assembled in the laboratory or sourced commercially (Table S3). The literature overwhelmingly used arrays assembled in the laboratory, including a variety of “custom” arrays and three “academic” arrays. Custom arrays, used by 62 studies, were relatively basic arrays arranged to fit the needs of a single study or of a group of studies conducted by a single research group. These arrays were typically composed of commercially available microphone elements mounted above the ground and attached by cable to a central multichannel recorder or laptop (e.g., Chen et al., 2006; Krakauer et al., 2009; Lapierre et al., 2011). Assembling these arrays requires familiarity with acoustic monitoring and audio hardware engineering in order to synchronize ARUs and record sounds. Typically, the construction of these arrays is not described in depth in the papers in which they are utilized. In contrast to “custom” systems, we define “academic” arrays as those for which the development of the array system itself was the subject of at least one academic paper. The four academic systems in the literature were more complex and had more features than one‐off custom arrays. Two of these “academic” systems were used in a limited number of studies. Calupca, Fristrup, & Clark (2000) described a recording system that was used in two acoustic localization studies (Hedwig et al., 2018; Thompson et al., 2009). Hutto & Stutzman (2009) described a system used in one study (Campbell & Francis, 2012). Most recently, Wijers et al. (2019) designed a recording system that has not yet been used in any additional papers. The fourth academic system, the VoxNet platform, was developed in a series of several academic papers. This system was initially called Acoustic ENSBox (Girod, Lukac, Trifa, & Estrin, 2006) and was eventually developed into a system called VoxNet (Allen et al., 2008). VoxNet included many of the desirable features of wildlife recording arrays (discussed next), such as robustness and self‐synchronizing capabilities. This platform was used in 12 acoustic localization papers (Ali et al., 2009; Ali et al., 2007; Cai, Collier, Girod, Hudson, et al., 2013; Cai, Collier, Girod, Lee, et al., 2013; Collier, Blumstein, et al., 2010; Collier, Kirschel, & Taylor, 2010; Harlow, Collier, Burkholder, & Taylor, 2013; Trifa, Girod, Collier, Blumstein, & Taylor, 2007; Vallejo & Taylor, 2009; Yu et al., 2016; Zhang et al., 2014). However, these arrays were not widely used outside of the research group that developed them, who noted their expensiveness and difficulty to maintain (Taylor, Huang, & Yao, 2016).

Commercial sources include both wildlife recording ARUs and general purpose ARUs. Wildlife recorders, commercially available ARUs built specifically for deployment in the field to record wildlife, were used in 15 studies (e.g., Kershenbaum et al., 2019; Spillmann et al., 2017; Suzuki et al., 2018). The most notable manufacturer of these ARUs is Wildlife Acoustics (Maynard, MA, USA), which sells ARUs that are ready to be deployed “out of the box” and include features such as programmable recording schedules, built‐in data storage and batteries, and waterproofing, making them ideal for long‐term autonomous deployment. Both Wildlife Acoustics and Frontier Laboratories (Brisbane, Australia), currently, sell wildlife recorders which synchronize automatically using GPS. Another wildlife recording system, the DACHO 16‐channel array used by Suzuki et al. (2018) and sold by System in Frontier (Tokyo, Japan), is apparently no longer available. Five studies performed DOA localization using general purpose, commercially available ARUs that were not originally designed for wildlife recording. These ARUs were built for other applications, such as clarifying sound during conference calls in an office setting. Examples include the Dev‐Audio Microcone (Suzuki, Matsubayashi, Hedley, Nakadai, & Okuno, 2017), which is no longer available, and the System in Frontier TAMAGO (Matsubayashi et al., 2017), which is commercially available at the time of writing.

#### 3.2.2 Number of ARUs and microphones

The minimum required number of ARUs and microphone elements per ARU varies by localization method and desired number of dimensions in which to localize. In general, localization accuracy improves as the number of microphones and ARUs used for localization increases, because averaging results from multiple ARUs reduces the influence of errors from any one recorder. For the purpose of this review, we consider an ARU to be a device constructed of one or several microphones that are rigidly attached to each other in one configuration. For instance, two microphones attached at the ends of a rigid plastic pole would be considered one ARU, whereas 16 microphones attached by nonrigid cable to a central recorder are considered separate ARUs, although they are physically connected. This definition usefully divides the methods used in the literature into three categories. First, most applications localizing audible sound of animals, such as birds or large mammals, use multiple widely spaced, single‐microphone ARUs. Second, most hyperbolic localization of ultrasonic bat vocalizations is achieved over small areas using a single multiple‐microphone ARU. Third, direction‐of‐arrival localization is achieved using one or more multiple‐microphone ARUs.

In hyperbolic applications which find the coordinate location of a sound, four microphones are required to unambiguously position a sound on a plane, and five are required for unambiguous positioning in three‐dimensional space (Spencer, 2007; Spiesberger, 2001). The required number of microphones was a common point of confusion in the literature reviewed. Much of the literature claimed that only three microphones and four microphones are required, respectively. This smaller number of microphones is sufficient to localize sound sources originating at certain positions with respect to a given microphone setup, especially sources closer to the center of the array. However, for some areas closer to the microphones, the TDOAs produced at one source position are identical to those produced at another source position. The distances between these positions vary from slight (0–1 m) to large (10–100 m; Spiesberger, 2001). For examples of this ambiguity and an intuitive explanation, see Spiesberger (2001).

Direction‐of‐arrival localization requires three microphones per ARU for two‐dimensional DOA estimation and four for three‐dimensional localization (see Section “Direction of arrival (DOA) localization”). Two or more ARUs each calculating a DOA can be used to recover a coordinate location by intersecting their DOA estimates. Ambiguous DOA solutions can occur when using fewer than the recommended number of microphones, but other information about the source's position can be used to eliminate uncertainty. For instance, Bates et al. (2010) recorded frogs using two ARUs that each had two microphones. This setup alone would not have identified a coordinate solution, but the animals were known to be calling from the surface of a pond on one side of the ARUs, allowing a unique solution to be found by process of elimination. Additionally, each microphone arrangement exhibits a trade‐off between precision and accuracy at certain frequency ranges (see Section “Placement”). Adding more microphones to an array at a given spacing increases precision without sacrificing accuracy (Chen et al., 2006). This approach could also improve the flexibility of the ARU for localizing sounds across a variety of frequencies, by using subsets of microphones from the ARU to create “subarrays” with different spacings (e.g., Kwan et al., 2006). Certain noise reduction techniques require that the number of microphone elements is greater than the number of active sound sources (e.g., Suzuki et al., 2017). Several applications used ARUs containing more than the minimum number of microphones (e.g., a 16‐microphone setup, Suzuki et al., 2018). Although both hyperbolic and DOA localization employ multimicrophone ARUs, microphone placement and spacing differ between these two applications (see Section “Placement”).

#### 3.2.3 Synchronization

Synchronization is the process of temporally aligning recordings from multiple microphones. This process is necessary in order to accurately measure the slight delays in arrival time of a sound at each microphone within the array. Even recorders that begin recording simultaneously will fall out of synchronization eventually if not periodically resynchronized. This tendency toward asynchrony is known as *drift* and occurs due to slight differences in true sampling rates of recording hardware. The amount of drift a recorder experiences varies by the quality and age of the recorder's internal oscillator (Guggenberger, Lux, & Böszörmenyi, 2015). Clock drift for ARUs may be on the order of 1 to 10 s per day (Clark et al., 2018; Thode et al., 2007).

The synchronization accuracy necessary depends on the type of localization performed. Asynchrony between two microphones is roughly equivalent to an inaccurate measurement of one of the microphone's positions. For example, consider a microphone with an internal time 1 ms ahead of the internal time of another microphone. Sound can travel about 0.3 m in that time, so this inaccuracy is equivalent to the first microphone being measured 0.3 m closer to the sound source than its true location. A 0.3 m error is small relative to the size of a widely spaced hyperbolic array, so a synchronization error <1 ms is likely sufficient for this application (Mennill et al., 2012). In contrast, 0.3 m is large relative to the microphone spacing of a DOA array, so more precise synchronization is required. This level of precision is attainable on closely spaced DOA microphones using cable synchronization.

Synchronization may occur during recording, for example, by connecting microphones via cable to a multichannel recorder and storage device such that all sound files are recorded simultaneously. Synchronization may also occur after field recording, in which case each individual ARU usually includes its own dedicated storage and recording device. This is the case for acoustic synchronization, in which a sound is played while ARUs are recording and is used to align audio recordings on a computer after the fact. Synchronization methods include cable synchronization, acoustic synchronization, GPS synchronization, or network communication between recorders. In one case, ARUs were not synchronized (Suzuki et al., 2018).

Cable synchronization involves connecting ARUs or microphones to a central multichannel recorder or computer. This process is straightforward for microphone arrays deployed across small spatial extents (e.g., Wang et al., 2005), or multimicrophone ARUs used for DOA localization or hyperbolic localization of bats (e.g., Hulgard, Moss, Jakobsen, & Surlykke, 2016; Kojima et al., 2016). Running cables over large areas can be impractical or impossible (Mennill et al., 2012).

One alternative, typically used for arrays of small spatial extent, is acoustic synchronization. This method involves playing back an artificial sound from a known location, computing based on this location the expected delay of the playback's arrival time at each microphone, then aligning recordings to these expected delays post hoc. Like other methods of synchronization, recorders must be synchronized frequently to avoid drift, meaning that this synchronization requires either frequent manual labor or an automated playback method. One group gathered all ARUs in one location before each night's deployment, played a synchronization sound heard on all recorders, and then walked the recorders to their deployment positions (Frommolt & Tauchert, 2014). Another application used a 3D‐printed holster to position an earphone, which played a quiet sound, at a known distance from microphone elements in the multimicrophone ARU (Hedley et al., 2017).

For arrays covering larger areas, GPS synchronization is a feasible alternative to cable and acoustic synchronization. Although GPS (global positioning system) is named for its ability to geolocate a GPS receiver, GPS satellites also provide time information to receivers. This approach involves attaching a GPS receiver to each ARU, then using the received GPS timestamps to align recordings and correct drift in the signal either in real time or in processing after field recording. Forested areas with thick canopy cover may hinder GPS receivers from establishing a reliable GPS fix (Huetz & Aubin, 2012). Wildlife Acoustics developed two recorders with GPS synchronization capabilities, the Song Meter SM2 and SM3, which were used in the literature reviewed but were recently replaced with the Song Meter SM4‐TS (Wildlife Acoustics, Maynard, MA, USA), but these ARUs are no longer manufactured. Another GPS‐synchronized ARU, the BAR‐LT (Frontier Laboratories, Brisbane, Australia), is currently available but was not used in the literature reviewed here.

Finally, recorders may synchronize by connecting to a shared wireless network. In the VoxNet array system, each ARU was controlled by a computer connected to a shared Wi‐Fi network, allowing for time synchronization in dense forests where GPS synchronization was unreliable (Harlow et al., 2013).

While synchronization between the microphones of an ARU is always necessary, DOAs arising from multiple ARUs can be intersected without precisely synchronizing the ARUs. When microphones record for only a short period of time, synchronization may not be necessary, as long as the time difference between sounds is much larger than the recorders' drift and all arrays localize the same sound (Cobos et al., 2017; Suzuki et al., 2018). However, microphones left to record autonomously for multiple hours or days will drift to the point of needing synchronization.

### 3.3 Field deployment

Variables to consider when deploying recorders in the field are properties of the acoustic recordings to be captured, placement of microphones and ARUs, and measurement of microphone and ARU positions (Figure 1). Field deployment typically requires multiple people and can be time‐consuming, especially at larger scales and with many ARUs. Ethier and Wilson (2019) reported that two people required 1–2 hr to deploy four GPS‐synchronized ARUs that were separated by 40 m.

In addition to deploying the microphone array, practitioners must measure the local temperature in order to accurately estimate speed of sound. During longer deployments, temperature and other climactic values such as humidity and wind speed may be obtained from a weather logger (e.g., Hennigar et al., 2019; Wahlberg et al., 2003) or nearby public weather stations. Many other preparations are required for setting up ARUs in general, such as selecting a power source and choosing a bit depth for recordings, but we do not focus on these here. For more information on these aspects of recorder deployment, see Blumstein et al. (2011).

#### 3.3.1 Recording properties

Two important features of acoustic recordings generated by ARUs are the sample rate at which they were recorded, and the duration and scheduling of each recording. Sample rate determines the maximum sound frequency able to be recorded and can influence the accuracy of position estimation. A microphone captures audio by transforming the vibrations from sound waves into a continuous voltage signal. Digital audio is recorded by sampling the value of the voltage signal, usually at a sample rate of thousands of Hertz (thousands of samples per second). To record a sound at any given frequency, the sample rate must be at least twice as high as the desired frequency, a minimum sample rate known as the *Nyquist rate*. Sounds at frequencies higher than half the Nyquist rate will be aliased into the audible frequencies, unless filtered out before recording. For birds, which often vocalize at frequencies below 10 kHz, a sample rate of 22.05 kHz or larger is commonly used. Sounds emitted at higher frequencies than an adult human can hear, typically above 20 kHz, are considered “ultrasound,” in contrast to sounds emitted below this threshold, which are referred to as “audible.” Ultrasonic bat vocalizations were recorded using sample rates as high as 500 kHz (Holderied, 2006). Some bat localization studies found that increasing sample rate may improve the precision with which time delays can be measured, increasing the accuracy with which sounds can be localized (e.g., by 10 cm, Surlykke et al., 2009). The effects of sample rate on position estimation accuracy have not been studied for most applications of terrestrial wildlife localization and merit further examination. However, the accuracy improvements may be negligible compared to other sources of error. The primary disadvantages of high sample rates are the specialized recorders required to capture them and the recordings' much larger file size.

Typically, studies involving ARUs record selectively instead of continuously. Recording schedule refers to the length of recordings to make, the time of day to record, and the dates to record. Selecting a recording schedule requires weighing the trade‐offs in the context of the research question. Longer recordings capture more biological information, but use more storage and battery power, thus shortening the amount of time they can be deployed autonomously. Additionally, longer recordings require more human labor to analyze, unless recording analysis is automated. Many ARUs, such as Wildlife Acoustics recorders and the AudioMoth, can be programmed to record at a certain time of day, which can be useful for selectively recording species when they are highly acoustically active. For instance, Wilson and Bayne (2018) programmed acoustic recorders to record birds between 5:30 a.m. and 8:30 a.m., as birds are particularly active around dawn. Alternatively, we suggest that a recording schedule could be chosen to limit the density of sounds in the soundscape, as a large number of overlapping vocalizations can hinder the performance of localization pipelines (e.g., Hedley et al., 2017; Simmons, Simmons, & Bates, 2008). Finally, recording dates must be chosen with species habits in mind as many animals are migratory, exhibit behaviors only seasonally, or are less vocal at certain times of year.

#### 3.3.2 Placement

Arrays for audible‐sound hyperbolic localization, ultrasound hyperbolic localization, and DOA localization differ in their placement requirements. Placement includes both distance between and geometric arrangement of equipment, and must be considered both for individual microphone elements within multimicrophone ARUs and for the ARUs themselves. Microphones for hyperbolic localization must be positioned close enough that each sound is recorded on at least four microphones for two‐dimensional localization or five microphones for three‐dimensional localization (see Section “Number of ARUs and microphones”). Researchers should decide the area in which animals will be localized, estimate the maximum distance a target sound can travel before it loses signal strength, and ensure that for any point in the localization area, a sufficient number of microphones are within this maximum distance. For accurate hyperbolic localization, microphone spacing must also be large enough that the animal's distance from the array is approximately the same order of magnitude as the distance between the microphones (Koblitz, 2018). Because of the differing acoustic properties of audible and ultrasonic sound, audible sound is localized on multiple widely spaced single‐microphone ARUs, whereas arrays for hyperbolic localization of bats are often composed of a single multimicrophone ARU. Direction‐of‐arrival localization always requires ARUs that contain multiple closely spaced microphones.

Hyperbolic localization of nonultrasonic sound involves multiple ARUs, each usually containing one microphone, with many variations in spacing and geometric arrangement (except a single ARU approach used to study individual perched birds, by Patricelli, Dantzker, et al., 2007; Patricelli, Dantzker, et al., 2008). Although multiple ARUs are used, these ARUs may be physically connected by cable (see Section “Synchronization”). Animals that make louder and less directed sounds can be localized on ARUs with wider spacing. The choice of distance between ARUs in hyperbolic arrays is influenced by the acoustic properties of the habitat and the study species; for instance, wolf howls can be heard from large distances so can be localized by arrays with larger spatial extent. Multi‐ARU arrays had a median spacing between ARUs of about 31 m (Table S1). The maximum area surveyed by any one array was 30 km^2^ in a test of a system intended for localizing wolf howls (Papin et al., 2018). The area enclosed within the boundaries of the microphones is sometimes referred to as the “hull” of the array. The hull of the array may be larger or smaller than the area in which researchers choose to localize animals. This was the case in the study using the second‐largest array, which enclosed a 3 km^2^ area but was used to localize orangutans vocalizing within a 4.5 km^2^ area within and surrounding the array's boundaries (Spillmann et al., 2017). As accuracy is worse outside the hull of the array (Bower & Clark, 2005; Kershenbaum et al., 2019; McGregor, Dabelsteen, Clark, Bower, & Holland, 1997), the most effective two‐dimensional geometric placement of microphones is a circle (e.g., Campbell & Francis, 2012), which maximizes the area within the hull. In four‐microphone arrays, a circular arrangement is typically accomplished by arranging microphones in a square (e.g., Grafe, 1997; Payne, Thompson, & Kramer, 2003). A grid may be used for localizing multiple individuals simultaneously or assessing large areas (e.g., Fitzsimmons, Foote, Ratcliffe, & Mennill, 2008b; Wilson & Bayne, 2018). Other geometric arrangements of ARUs in audible‐sound arrays included polygons (e.g., Spiesberger, 1999; Thompson et al., 2009) and T‐shaped arrangements (Magyar, Schleidt, & Miller, 1978). Accurate three‐dimensional hyperbolic localization of audible sound requires large vertical separation of the microphones (Spiesberger, 1999). For instance, one system tested for monitoring the three‐dimensional position of nocturnally migrating birds used three ARUs positioned at the points of an equilateral triangle, each ARU containing two microphones vertically separated by 7.5 m, to form a triangular prism (Stepanian et al., 2016). Ethier and Wilson (2019) tested a similar array of four ARUs positioned at the points of a 40 m × 40 m square, with each ARU containing two microphones separated vertically by 2–3 m, but found that this vertical separation was insufficient for accurate vertical position estimation. Only four studies achieved three‐dimensional positioning of audible sound using hyperbolic localization (Harlow et al., 2013; Hennigar et al., 2019; Spiesberger, 1999; Stepanian et al., 2016).

The primary concern in hyperbolic localization of ultrasonic bat vocalizations is the distance between and geometric arrangement of microphones in a single ARU, as most studies used one or multiple multimicrophone ARUs (but see Jensen & Miller, 1999; Roeder, 1966). The ultrasonic, highly directional calls of bats preclude the use of widely spaced ARUs, as high‐frequency sounds attenuate in the atmosphere more quickly than low‐frequency sounds, so ultrasonic vocalizations cannot be heard from as far a distance (Koblitz, 2018). Of the 26 ultrasound localization studies using multimicrophone ARUs, 16 used a single ARU containing multiple microphones separated by about 1 m (range 0.2–2.58 m, Table S1). Multimicrophone ARUs for bats were arranged in a variety of geometries, as reviewed by Koblitz (2018), including T‐shaped (e.g., Brinkløv, Kalko, & Surlykke, 2010; Götze, Koblitz, Denzinger, & Schnitzler, 2016; Kounitsky et al., 2015), linear (e.g., Surlykke & Kalko, 2008; Surlykke et al., 2009), and grid (Seibert et al., 2013, 2015). In the 12 studies using multiple ARUs, ARUs were used individually to track a bat's path over a large area (e.g., a 15 m × 22 m area, Fujioka, Aihara, Sumiya, Aihara, & Hiryu, 2016; Fujioka et al., 2014; Motoi et al., 2017; Sumiya et al., 2017), or were used simultaneously (e.g., Goerlitz et al., 2010; Holderied & Helversen, 2003). The spacing between these ARUs ranged from 5.5 m to 22 m, and the array which covered the largest area localized animals within 25 m of the array (Table S1).

As in bat localization, the most important considerations in DOA array design were the distance between and geometric arrangement of microphones. A single DOA ARU does not localize sounds equally well across all frequencies. The frequency range across which it localizes most precisely and accurately is determined by the spacing between the microphones in the array (Ali et al., 2007; Trifa, 2006). If the wavelength of a sound is less than twice the spacing between microphones within an ARU, the DOA may be estimated inaccurately, especially in noisy environments. However, when the wavelength is greater than twice the spacing between microphones, DOA estimates become less precise (Trifa, 2006). Thus, high‐frequency sounds, which have smaller wavelengths, require smaller spacing between microphones. The intermicrophone distance for DOA arrays was between 3 and 12 cm, except in one study which compared an ARU with 61 cm microphone spacing to a more typical 4 cm intermicrophone distance (Wang et al., 2005). Multimicrophone ARUs for DOA localization were often arranged in more complex three‐dimensional geometries than the multimicrophone ARUs used for bat localization (but see Bates et al., 2010). Examples include a ring of microphones with one microphone above the plane of the ring (Suzuki et al., 2017) or four microphones positioned at the corners of a tetrahedron (e.g., Voxnet, Cai et al., 2013). Two or more multiple‐microphone ARUs spaced widely enough apart can localize the sound by intersecting the DOAs estimated by the ARUs. Arrangements for DOA ARUs included pairs (e.g., Simmons et al., 2008), polygons (e.g., Suzuki et al., 2018), and squares (e.g., Wang et al., 2005). The spacing between ARUs varied from 4 to 70 m (Table S1).

#### 3.3.3 Microphone position measurement

Localization depends on a precise knowledge of the microphones' relative positions, which can be derived through direct measurement, GPS locations, or acoustic self‐survey. Similar to synchronization accuracy, the smaller the distance between microphones, the more accurately their locations must be known (see Section “Synchronization”). Direct measurement is appropriate for measuring the smallest distances. Two common methods of direct measurement are using a measuring tape, for instance, to measure multimicrophone ARUs such as those commonly used for bats (Ing et al., 2016), and using surveying techniques, such as measuring distances with a laser rangefinder, for multi‐ARU arrays with a small‐to‐moderate spacing between microphones (Spiesberger, 1999). The dimensions of some manufactured or 3D‐printed arrays may be premeasured (e.g., Suzuki et al., 2016; Wijers et al., 2019). For arrays with a larger spatial extent, such as those used for localizing songbirds, survey‐grade GPS receivers with meter‐ or centimeter‐level accuracy are effective (e.g., Mennill et al., 2012; Wilson & Bayne, 2018). A limited number of studies estimated microphone position using GPS receivers onboard the ARUs (e.g., Kershenbaum et al., 2019; Spillmann et al., 2015). GPS receiver measurements are more accurate when averaged over time. The accuracy of these measurements is sufficient for arrays with very large spacing, such as those used to localize wolves or monitor for gunshots, but may be insufficient for other applications. Lastly, an acoustic self‐survey technique allowed Acoustic ENSBox and VoxNet recorders to automatically solve for microphones' relative positions and orientations using ranging chirps (Allen et al., 2008; Girod et al., 2006).

### 3.4 Sound processing

After a field deployment, recordings may be processed in several ways prior to position estimation, including noise reduction, sound detection, and calculation of TDOAs (Figure 1). Over the course of the deployment, the microphone array has recorded a series of soundscapes, defined as the combination of all biological, geological, and anthropogenic sound present in an environment at a given time (Pijanowski et al., 2011). These recordings contain both the target sounds to be localized and extraneous sounds such as noise from wind, vehicles, and other species. Noise reduction eliminates background noise before or after detecting sounds. Sound detection involves identifying a set of individual sounds to localize from within longer recordings. Finally, many localization methods require explicit calculation of sound TDOAs. These three processing steps may be performed by a combination of automated and manual methods.

Table S2 describes software used for sound processing in the literature reviewed, but many other bioacoustics signal processing techniques are available. For instance, some recently released programs such as Acoular (Sarradj & Herold, 2017), warbleR (Araya‐Salas & Smith‐Vidaurre, 2017), and AviaNZ (Marsland, Priyadarshani, Juodakis, & Castro, 2019) were not used in the literature reviewed here, but we anticipate their use will be helpful for future practitioners of acoustic localization. For a more complete listing of sound processing techniques and software packages, see Priyadarshani, Marsland, & Castro (2018).

#### 3.4.1 Noise reduction

Noise reduction, the reduction of background noise and nontarget sounds, can improve sound detection performance, TDOA calculation accuracy, and classification performance. Of the 95 studies reviewed, 46 reported using a noise reduction technique. Both frequency filters and sound source separation were used to reduce background noise, such as wind or sounds from nonfocal species. Frequency filters may be analog or digital and include low‐pass filters to remove high‐frequency sounds, high‐pass filters to remove low‐frequency sounds, and band‐pass filters to remove sound in frequencies outside of a particular desired band of frequencies. Analog filters are physical circuits that exclude frequencies before the sound is saved to the recording device. Digital filters are algorithms, such as those implemented in MATLAB or acoustic analysis software, that can be applied before detecting sounds, after detecting sounds, or as a component of the localization algorithm (e.g., MUSIC, Suzuki et al., 2017). Either method of filtering requires a priori knowledge of the frequency band used by the species being studied. Alternatively, some systems separate sounds from noise using a process called *beamforming*. This process requires closely spaced microphones that record very similar signals, so is primarily used alongside DOA‐based applications (e.g., HARKBird, Suzuki et al., 2017), but one paper performed source separation using a hyperbolic localization array that covered a small (9 m^2^) area (Jones & Ratnam, 2009). Other digital sound reduction techniques are available, such as algorithms that use samples of pure background noise to identify and remove noise (e.g., Audacity Team, 2019; Boll, 1979) and a method that uses a combination of frequency filtering and wavelet decomposition of the signal (Priyadarshani, Marsland, Castro, & Punchihewa, 2016). The relative efficacy of noise reduction techniques is reviewed by Priyadarshani, Marsland, et al. (2018).

#### 3.4.2 Sound detection

Sound detection involves sifting through extraneous sounds present in the soundscape to identify the set of sounds to be localized. Sound detection can be performed either using manual or semi‐automated methods, the former being more common. Of 63 studies that reported how sounds were detected, 39 performed detection entirely manually, 6 reported completely automatic detection without manual review, and 15 required manual intervention or review of automatically detected calls. Another aspect of sound detection is the classification of the species or individual producing the call, which can be performed using manual or automated methods. A variety of software, such as Kaleidoscope (Wildlife Acoustics, Maynard, MA, USA) and HARKBird (Suzuki et al., 2017), was used for the sound detection and classification process in the literature reviewed (Table S2), and even more techniques are now available (Priyadarshani, Marsland, et al., 2018).

Sounds were manually detected by listening to audio or inspecting spectrograms in software like Audacity (Audacity Team, 2019) and Raven (Charif, Waack, & Strickman, 2010). Manually identified detections are sometimes considered to represent ground truth or the highest level of accuracy (e.g., Spillmann et al., 2015; Suzuki et al., 2016), but in several instances, automated detection methods identified faint vocalizations that were missed during manual review (Ethier & Wilson, 2019; Suzuki et al., 2017). Manual detection may be time‐consuming. Human review of a 10 min recording can take 20–30 min (Celis‐Murillo, Deppe, & Allen, 2009; Hutto & Stutzman, 2009), although this is likely to vary with the complexity of the soundscape and purpose of the experiment. Some studies employed human observers in the field to note sounds or behavioral events 584 to localize (e.g., Collier, Blumstein, et al., 2010). In practice, manual review generated detections numbering in the dozens (e.g., Lippold et al., 2008; Mennill & Vehrencamp, 2008) to thousands (Hennigar et al., 2019). One aquatic localization study reported detecting over 22,000 sounds manually (Clark, Charif, Mitchell, & Colby, 1996).

Automated methods used for sound detection included a variety of amplitude‐based triggering methods (e.g., Bates et al., 2010; Collier, Blumstein, et al., 2010; Eastman & Simmons, 2005), machine learning (Spillmann et al., 2015, 2017), template matching or cross‐correlation (e.g., Araya‐Salas et al., 2017; Frommolt & Tauchert, 2014), and MUSIC (MUltiple SIgnal Classification) methods (e.g., Suzuki et al., 2017, 2016). Automated methods may produce false negatives, where the method does not detect all relevant vocalizations, and false positives, where the method detects nontarget sounds. The accuracy of these methods varies widely. Suzuki et al. (2018) used manual review to assess the performance of MUSIC methods, which simultaneously detect and localize sounds. The MUSIC method generated approximately 3%–27% false positives and 0%–50% false negatives, depending on the individual animal localized and the session in which the sound was recorded. Ethier and Wilson (2019) used an amplitude triggering method implemented in Kaleidoscope (Wildlife Acoustics, Maynard, MA, USA). This study reported that <1% of automated detections were false positives. Comparing this detection method to manual review of a 2‐hr long recording, Ethier and Wilson (2019) found that the automated method successfully detected all manual annotations as well as picking up some detections that were too faint to be annotated by manual review. Automated methods are attractive due to their scalability to many hours of recording (Darras et al., 2019; Marsland et al., 2019), but calibration of these methods can be time‐consuming and may require expert knowledge (Priyadarshani, Marsland, et al., 2018). The number of detections generated by automated methods was as many as 2.7 million (Ethier & Wilson, 2019).

Automated methods may either be used alone or paired with manual review of detections. Six studies reported completely automated sound detection. Of these, three methods used the MUSIC algorithm, which requires an estimate of the number of sound sources to be detected (Kojima et al., 2016, 2017; Suzuki et al., 2016). The three remaining methods leveraged amplitude information to detect vocalizations, including an amplitude threshold within the frequency band of the target species (Simmons et al., 2008), a system that discarded extraneous low amplitude wind noise and used template matching to identify vocalizations in the remaining audio (Wang, Elson, Estrin, & Yao, 2003), and an amplitude‐detecting algorithm capable of adapting to continuously changing noise levels (Trifa et al., 2007). Curation methods for automated detectors included manually identifying calls that were not detected by automated detectors (e.g., Hügel et al., 2017) and removing false‐positive detections (e.g., Ali et al., 2007; Araya‐Salas et al., 2017). Another common manual curation step was excluding undesirable sounds from the target species, such as vocalizations with poor signal‐to‐noise ratios (e.g., Mennill et al., 2012; Papin et al., 2018; Sumiya et al., 2017) or vocalizations that were overlapped by the sounds of other species (e.g., Holderied, 2006; Krakauer et al., 2009). Some studies did not need to localize all sounds for species that moved infrequently, and selected a smaller set of sounds to be localized (e.g., Osmun & Mennill, 2011).

In addition to locating sounds to identify, sound detection may also require classification of sounds to identify species or individuals of interest. Manual reviewers classified species and individuals by the distinctive traits of their sounds (e.g., Spillmann et al., 2017; Suzuki et al., 2016) or by cross‐referencing against field observations (e.g., Krakauer et al., 2009). Several papers utilized automated methods for classification, including template matching (Wang et al., 2003) and machine learning (Vallejo & Taylor, 2009).

#### 3.4.3 Time delay calculation

After identifying sounds to localize, their relative arrival times at each microphone must be calculated either directly or implicitly. Direct calculation involves cross‐correlating the sounds' spectrograms or waveforms. Waveform cross‐correlation allows for more precise position estimation, but requires higher signal‐to‐noise ratio to detect signals from waveforms (Wilson et al., 2014). Cross‐correlation can be performed by bioacoustic analysis software such as Raven Pro and its predecessor Canary, XBAT, EarLab, AviSoft SASLab, and a variety of proprietary custom‐written programs such as SDEer, SigPro, and ArrayGUI (Table S2). A low‐tech method of finding these relationships is to visually identify the time at which the sound of interest starts. This manual identification can be performed using spectrogram‐inspection software like Raven Pro, or even by identifying impulses on a waveform by hand as by Roeder (1966). However, manually identifying onsets is time‐intensive and prone to error, as onset can occur over several milliseconds. Alternatively, TDOAs are calculated implicitly, not directly, by Correlation Sum algorithms (described in Section “Hyperbolic localization”) or in DOA algorithms.

### 3.5 Position estimation

After sound processing, position estimation algorithms are used to determine the sound source's location (Figure 1). Both hyperbolic and DOA localization approaches included several different algorithms. Algorithms were typically implemented in computer software, as listed in Table S2.

Many papers tested the accuracy of position estimation of their localization system using playback or live animal tests. Position estimation error was typically calculated as the distance between the true position of a sound and its position estimated by acoustic localization. One method to estimate this error for a given deployed array is to localize sounds that were played from a speaker at a known position. If possible, it is desirable to test the array in true field conditions by measuring the position of a vocalizing live animal using GPS or a laser rangefinder, then estimating the animal's position using acoustic localization. Accuracy and precision varied widely based on the method used and may be improved by a variety of manual and automated methods.

Position estimation error can arise in many steps of the localization process, including selection of the number of microphones, synchronization of microphones, estimation of speed of sound, placement of the microphones and the measurement of their positions, true position of the recorded sound source, calculation of TDOAs, and association of DOAs in DOA intersection applications. First, arrays using fewer than the recommended number of microphones have some areas of localization ambiguity (see Section “Number of ARUs and microphones”; Spiesberger, 2001), as do arrays with a “singular” arrangement (see Section “Placement”). Second, with a typical microphone drift on the order of 1–10 s per day (Clark et al., 2018; Thode et al., 2007), microphones must be tightly synchronized and resynchronized frequently, or else TDOA measurement will be inaccurate. This can pose problems in densely vegetated habitats where GPS synchronization or cable synchronization is more challenging (see Section “Synchronization”). Third, localization relies on accurate estimation of speed of sound, so inaccurate temperature measurements, or conditions such as high wind and humidity, can affect the accuracy of position estimates (McGregor et al., 1997; Spiesberger & Fristrup, 1990). Fourth, many aspects of microphone placement must be carefully selected for the study to maximize localization accuracy. Wider‐spaced hyperbolic arrays typically return less accurate localization results (Mennill et al., 2012), possibly due to the lower amplitude of the attenuated sounds reaching the microphone. For DOA applications, the intermicrophone distance within one ARU determines the trade‐off between precision and accuracy at a particular frequency (see Section “Placement”). Fifth, inaccurate measurement of microphone positions also causes errors, which can be thought of as roughly interchangeable with synchronization errors (see Section “Synchronization”). Sixth, the true position of the sound source with respect to the array matters: For a hyperbolic localization array, localization is more accurate inside of the boundaries of the array than outside of the array's hull and is more accurate closer to the center of the array than closer to its edges (see Section “Placement”; Bower & Clark, 2005; Kershenbaum et al., 2019; McGregor et al., 1997). Seventh, localization inaccuracy may arise from errors in calculating TDOAs, deriving from problems such as overlapping noise or nontarget sounds, attenuation and reverberation in forested habitats (Mennill et al., 2012), and cross‐correlation inaccuracies of sounds with little frequency modulation (Bower & Clark, 2005). Finally, in DOA intersection applications, the so‐called data‐association problem can cause inaccurate coordinate localization (see Cobos et al., 2017).

#### 3.5.1 Hyperbolic localization

The hyperbolic approach is the most commonly used in the literature. Of 86 papers reporting the position estimation algorithm used, 69 used algorithms in this category. Hyperbolic localization assumes that the sound waves radiate out spherically from the sound source. The sound wave arrives at each microphone at a slightly different time. Consider the sound's arrival time at two microphones. The difference in arrival time measured in seconds, multiplied by the speed of sound in meters per second, defines a distance in meters. This distance is the difference in how close the sound is to each microphone. For instance, if a sound arrives at microphone A 0.01 s before it arrived at microphone B, and the speed of sound is about 343 m per second, then the sound is 3.43 m closer to microphone A than microphone B. This difference in distance defines a set of potential locations that the source could have originated at. These locations form a contour that has the shape of a hyperbola in two‐dimensional space and a hyperboloid in three‐dimensional space. With the addition of more pairs of microphones, more contours are calculated. Ideally, their intersection gives the sound's location. When there is no perfect intersection due to the inaccuracies of localizing in noisy environments or reflective habitats, algorithms may estimate the point that minimizes the sum of the squared distances to the contours.

There are two approaches to hyperbolic position estimation: two‐stage and one‐stage (Svaizer, Matassoni, & Omologo, 1997). In the straightforward two‐stage approach, described in the previous paragraph, TDOAs are first calculated during the sound processing step, then input into position estimation algorithms to estimate the sound's coordinate location. This method, used in 49 studies, is often referred to as TDOA localization or time delay estimation localization. Some TDOA localization approaches involve calculating the shape of the contours and solving for their intersection (e.g., Surlykke & Kalko, 2008). Other two‐stage approaches used algorithms that calculate this intersection directly, without first calculating the shape of the contours (see Gillette & Silverman, 2008; Halverson, 2002; Militello & Buenafuente, 2007). The 20 remaining hyperbolic localization papers employed a one‐stage algorithm, which implicitly uses TDOA information without explicitly calculating TDOAs. The first use of these methods in terrestrial localization was the Correlation Sum algorithm described by Mennill, Fristrup, & Vehrencamp (2006). The Correlation Sum algorithm involves proposing potential source locations in relation to the GPS coordinates of each microphone. At each proposed source location, calculating the distance between the proposed location and the microphone, then dividing this distance by the speed of sound, produces an estimate of the difference in the arrival time of the sound at each microphone. The recordings are offset from each other at a range of time delays and cross‐correlated at each offset. In theory, the cross‐correlation should reach a maximum when the recordings are offset by the sound's true time difference of arrival. For each proposed source location, the value of the cross‐correlation functions is extracted at the estimated arrival times and summed. The position estimate is chosen to be the location for which this sum is the largest. This optimization procedure is similar to that of delay‐and‐sum beamforming, described in Section “Direction of arrival (DOA) localization” (Mennill et al., 2006). Similar algorithms based on the accumulated correlation method described by Bircheld (2004) were employed in three papers (Collier, Blumstein, et al., 2010; Collier, Kirschel, et al., 2010; Harlow et al., 2013).

Software options for hyperbolic localization are limited. We are not aware of any software that performs the entire two‐stage TDOA position estimation pipeline, and the software implementation of the one‐stage Correlation Sum algorithm was not published, although the method is described in detail by Collier (2010). Therefore, to employ hyperbolic localization, practitioners must currently either develop their own software or use separate software for each stage of two‐stage position estimation. For instance, Raven Pro can be used to compute TDOAs or estimate TDOAs visually by the relative time of arrival on spectrograms. These TDOAs can then be input into open‐source software such as Sound Finder to localize the animal (Wilson et al., 2014). Sound Finder, available in both R and Excel, uses the inverse of the algorithm used in global positioning systems, which does not require direct calculation of the shape of the contours (Halverson, 2002). Most other studies used unpublished custom scripts, usually written in MATLAB, to estimate locations via TDOA. One paper included in its appendix the MATLAB code used to calculate location via TDOA (Kershenbaum et al., 2019), and multiple papers used two named software packages that are not openly available online: ArrayGUI/ArrayBatchGUI (John Burt, Seattle, WA, USA) and SigPro (Simon Boel Pedersen). Canary, a commercial software package, was able to calculate TDOA and coordinate location simultaneously (e.g., McGregor et al., 1997), but it is no longer available.

Hyperbolic position estimation can be accurate to under a meter (e.g., Collier, Kirschel, et al., 2010; Grafe, 1997; Krakauer et al., 2009), especially if TDOAs are manually reviewed for accuracy (e.g., McGregor et al., 1997). An average error of 5 m is typical, and among hyperbolic localization systems that reported average accuracy, the median average accuracy was 2.12 m, although many studies reporting accuracy used methods to remove the inaccurate localizations, as discussed below. Localization is more accurate when spacing between ARUs is smaller (Wilson & Bayne, 2018), the habitat is open or fieldlike (McGregor et al., 1997), and the source is closer to the center of the array (Bower & Clark, 2005; Campbell & Francis, 2012; McGregor et al., 1997; Papin et al., 2018).

Several strategies were reported for reducing potential location error. Some hyperbolic localization algorithms estimated positional accuracy (e.g., Wilson et al., 2014). Some studies ignored position estimates that did not reach a predetermined threshold of accuracy (e.g., Mennill et al., 2012; Thompson et al., 2009; Wahlberg et al., 2003). Other studies accounted for position error by establishing an area for which position estimates were acceptably accurate, usually the area enclosed by the array or within a certain distance of the array. These studies then rejected sound source estimates that fell outside of the established limits (e.g., Foote, Fitzsimmons, Mennill, & Ratcliffe, 2008b; Fujioka et al., 2014; Spillmann et al., 2015; Spillmann et al., 2017; Surlykke et al., 2009). Other strategies checked to ensure that estimates corresponded with field observations (Surlykke et al., 1993), or estimated some aspect of the animal's position, such as direction or height, based on field observations instead of by acoustic localization (e.g., Jensen & Miller, 1999; Surlykke & Kalko, 2008). Correspondence with field notes or other data, such as photographs or videos, was also used as a rough measure of accuracy (e.g., Collier, Blumstein, et al., 2010; Eastman & Simmons, 2005; Jones & Ratnam, 2009; Spiesberger, 1999; Surlykke & Kalko, 2008).

#### 3.5.2 Direction of arrival (DOA) localization

The remaining 19 papers used DOA algorithms. The intuition behind DOA localization is similar to hyperbolic localization, except DOA localization makes a far‐field assumption, assuming the sound waves are planar. The difference in the sound's arrival time at two microphones, multiplied by the speed of sound, measures the additional distance the planar sound wave travels to the farther microphone. This distance is used to form a right triangle with the imaginary line connecting the two microphones, from which the direction of arrival can be calculated (see Figure 2b). For instance, if a sound arrives at microphone A 0.01 s before it arrives at microphone B, and the speed of sound is about 343 m per second, then the plane travels an additional 0.343 m to reach microphone B. If microphone A is 0.1 m from microphone B, then the angle formed by the wave is $\cos^{-1}\frac{0.1}{0.343}\approx 73$ . However, with two microphones alone, this angle does not describe with certainty the direction of arrival of the sound. Instead, the angle defines a symmetrical three‐dimensional cone, where the cone's axis is on the line formed by the two microphones. Given only input from two microphones, the sound source could have originated from any position on this cone. In the two‐dimensional case, where the sound is assumed to arrive from a particular plane, the uncertainty is limited not to a cone, but to two potential DOAs formed by the intersection of the plane and the cone. In this situation, adding another microphone eliminates one of the candidate DOAs. In the three‐dimensional case, two additional microphones create additional cones, which intersect to identify a single direction of arrival. Furthermore, DOA intersection methods allow for estimates of an animal's coordinate position using two or more multimicrophone ARUs: When multiple spatially separated ARUs find the DOA of the same sound, their intersection or center of gravity estimates the sound's coordinate location.

As in hyperbolic localization, direction‐of‐arrival techniques in the literature divided into two‐stage and one‐stage approaches. The previous paragraph describes a two‐stage approach, which involves the direct calculation of TDOAs. Among two‐stage approaches, two algorithms were used: a direct model of the human auditory system (Bates et al., 2010; Simmons et al., 2008) and an unspecified DOA approach (Schul et al., 2000). The seventeen remaining approaches were one‐stage methods, which do not require explicit calculation of TDOA. Eleven papers used a beamforming approach known as approximate maximum likelihood (AML). This method is similar to the Correlation Sum method for hyperbolic localization described in Section “Hyperbolic localization” in that both search across a range of possible position estimates to find the best estimated coordinate position (Correlation Sum algorithm) or DOA (AML algorithm). Unlike the Correlation Sum method, AML involves a far‐field assumption and weights sensor data based on the amplitude of the received signal (Chen, Hudson, & Yao, 2002). One approach called FD‐DOA estimated DOA without a search (Yu et al., 2016). Lastly, six papers used MUltiple SIgnal Classification (MUSIC, e.g., Hedley et al., 2017; Suzuki et al., 2016). This algorithm differs from the previous two approaches in that it does not directly use time delay information from the raw signal, but instead calculates a spectrogram first (Suzuki et al., 2017).

Direction‐of‐arrival methods are implemented in three open‐source software packages. The open‐source packages HARKBird (Suzuki et al., 2017) and SDEer (Hedley et al., 2017) implement the MUSIC algorithm with graphical user interfaces. HARKBird is written in Python and builds upon another program for DOA estimation, HARK (Nakadai et al., 2010) SDEer is a set of scripts written in MATLAB. Simmons et al. (2008) used a third software, EarLab, a MATLAB software that estimates DOA using a model of binaural hearing. No study published the scripts implementing the widely used AML algorithm.

Accuracy of DOA methods for terrestrial wildlife localization is not well established due to the limited number of DOA systems, but DOA intersection methods seem to perform comparably to hyperbolic localization systems. For example, Suzuki et al. (2018) reported position error of 5.5 ± 4.5 m (mean ± *SD*) for continuously observed vocalizing birds. The best‐performing DOA intersection system was a VoxNet array, which demonstrated position error of 0.199 ± 0.064 m (mean ± *SD*) for a playback experiment and 0.455 ± 0.500 m (mean ± *SD*) for localization of live birds (Collier, Kirschel, et al., 2010).

The accuracy of these methods was improved by manually or algorithmically excluding the estimated positions of noisy or poorly localized sounds. Methods included manually removing noise (Ali et al., 2007; Suzuki et al., 2017) and removing sounds with low signal‐to‐noise ratios (Bates et al., 2010). Ali et al. (2007) also removed all recordings created by two malfunctioning recorders, which were thought to have poor accuracy due to reverberation from nearby trees. Suzuki et al. (2018) used three ARUs to independently estimate DOAs to vocalizing birds every 0.2 s. ARUs did not always localize the same sound, resulting in a challenge known as the *data‐association problem* (see Cobos et al., 2017). These mismatched sound localizations were excluded from further analysis by an algorithm that required the beginning and end of each sound source to match up, and that the intersections of each of the three DOAs were within 15 m of each other.

## 4 DISCUSSION

### 4.1 Current literature

Our review highlights three unique aspects of the localization literature: the eight purposes of localization, the strengths and weaknesses of the two broad methods of localization, and the widespread requirement for human intervention in the localization process.

We identified eight distinct purposes in ecology and animal behavior for localization systems: assessing individual animals' positions or movements, localizing multiple individuals simultaneously to study their interactions, determining animals' individual identities, quantifying sound amplitude or directionality, selecting subsets of sounds for further acoustic analysis, calculating species abundance, inferring territory boundaries or habitat use, and separating animal sounds from background noise to improve species classification. Without localization, ARUs have limited ability to address these questions. Arrays of nonsynchronized microphones can assess differential habitat usage, but only across large scales. Some preliminary work has attempted to estimate animal density using information about call rate or amplitude captured in ARU recordings. Call rate methods determine the average sound production rate for a species, identify the sound production rate on a given acoustic recording, and then use these quantities to estimate density of the sound‐producing animals (Stevenson et al., 2015). Amplitude‐based approaches estimate the number of calling animals by leveraging the fact that the farther an animal is from a microphone, the lower its sound amplitude will be. For instance, two vocalizing animals, one closer to the microphone than the other, can be distinguished on a single‐microphone recording based on differences in the amplitude of their sounds. These methods may be inappropriate for large‐scale studies due to their need for calibration or review, including human interpretation to distinguish individuals (e.g., Celis‐Murillo et al., 2009; Darras, Furnas, Fitriawan, Mulyani, & Tscarntke^2018^; Dawson & Efford, 2009) or acoustic calibration specific to habitat, species, or recorder type (e.g., Darras et al., 2018; Yip, Leston, Bayne, Sólymos, & Grover, 2017). However, it is possible to use localization to calibrate these indices (Thompson et al., 2009).

There is no single best practice for acoustic localization of wildlife, but rather a suite of decisions that depend on the particular needs of the study (Table 2). In particular, hyperbolic and direction‐of‐arrival (DOA) localization each has unique strengths and weaknesses, as well as some areas in which their performance is comparable. Hyperbolic localization dominated the literature, making up about 77% of the studies, and has advantages over DOA localization in the usability of commercially available recorders, the ease of designing “custom” ARUs, applicability of an array to sounds at a wide range of frequencies, and intuitiveness of the localization algorithm. First, commercially available hyperbolic ARUs have simpler hardware than DOA recorders and often come in a waterproof housing with a screen or user interface, unlike the currently commercially available DOA devices (see Section “Recorder source”). Second, a relatively inexpensive “custom” hyperbolic array can be constructed by attaching microphones via cable to a central recorder, such as a Zoom F4 Multitrack Field Recorder (550USD, Zoom North America, Hauppauge, NY). In contrast, the exacting requirements for spacing and positioning of microphones in DOA ARUs make them less amenable to custom design. Third, a single hyperbolic array is applicable to sounds produced in a wide range of frequencies, whereas DOA ARUs localize most precisely and accurately at a band of frequencies determined by the spacing between the microphones in the array (see Section “Number of ARUs and microphones”; Ali et al., 2007; Trifa, 2006). Last, the more intuitive hyperbolic localization algorithm and the greater time delays associated with hyperbolic localization may allow users of this method to more easily manually review localization results (see Section “Hyperbolic localization”).

**Table 2:** Considerations for method design of hyperbolic and direction‐of‐arrival (DOA) localization

Step	Substep	Considerations
1. Research question	1. Purpose	Direction of arrival (DOA) is sufficient for some purposes, but most require coordinate location
		Different purposes require different levels of localization accuracy
	2. Target animals	Acoustic overlap between study species and background noise complicates processing
	3. Spatiotemporal scale	Monitoring applications require longer study duration
2. Hardware	1. Recorder source	No synchronizing autonomous recording units (ARUs) are commercially available
		Hyperbolic and DOA localization require different array designs
	2. Number of ARUs/mics	At least 4 microphones are required for unambiguous localization
		Ambiguous locations may be acceptable in certain contexts
		DOA performance may be improved by using more microphones
	3. Synchronization	Cable synchronization is challenging for large spatial extents
		Dense canopies may prevent GPS synchronization
3. Field deployment		Temperature must be logged to accurately estimate speed of sound
	1. Recording properties	Sampling rate must be ≥2× the highest frequency to record
		Higher sampling rates allow recording of higher‐frequency signals, but require more storage
	2. Placement	Closer microphone positioning is required to record quiet, highly directional, and high‐frequency sounds on a minimum number of ARUs
		Optimal within‐ARU spacing for DOA microphones is half the sound wavelength
	3. Position measurement	Smaller microphone spacing requires more accurate positioning
		Survey‐grade GPS or acoustic self‐survey can be used for arrays of many ARUs separated at large distances
4. Processing	1. Noise reduction	Must reduce amplitude of background noise and nontarget species
		DOA methods may automatically reduce noise from other species
	2. Sound detection	Sound detection can be automated but requires manual review
		DOA methods may automatically detect sounds
	3. Time delay calculation	Background sounds may reduce accuracy of delay calculation
		Some methods do not require explicit calculation of delays
5. Position estimation	1. Hyperbolic	Algorithm is less robust to background noise
	2. Direction of arrival	Some algorithms can effectively reduce background noise
		DOA estimates can be combined to estimate coordinate location
Publishing results	Design	Report ARU and microphone geometry and spacing, and accuracy of ARU position estimates
		Report manual and automated processing and localization methods
		Report hours of human labor and/or computational time used for each step of localization
		Publish implementations of or links to software
	Performance	Summarize recall and precision of automated sound detectors
		Summarize accuracy and precision of position estimates
		Report performance with and without manual curation

Direction‐of‐arrival localization outperforms hyperbolic localization in terms of cost of commercially available arrays, ease of recorder deployment for three‐dimensional localization of audible sounds, production of noise‐reduced recordings, automated sound detection, and software availability. First, a DOA microphone array is commercially available for 100USD, though it must be attached to a laptop to create an ARU (ReSpeaker, Seeed Technology Co. Ltd., Shenzhen, China). Another DOA ARU can be fabricated for under 200USD per ARU (CARACAL, Wijers et al., 2019). A minimum of two DOA ARUs can then be used to obtain two‐dimensional coordinate localization. In contrast, the price of an individual two‐microphone wildlife recorder for hyperbolic localization exceeds 800USD (Wildlife Acoustics, Maynard, MA, USA), and at least two of these ARUs must be used to meet the minimum four microphones required for two‐dimensional hyperbolic localization. Second, direction‐of‐arrival localization is easily applied to three‐dimensional localization of audible sounds by intersecting three‐dimensional DOA estimates from two or more ARUs, which need not be vertically separated (e.g., Harlow et al., 2013). Field deployment of microphones for three‐dimensional hyperbolic localization of audible sounds is more challenging, as this application requires that microphones be separated vertically by several meters (see Section “Placement”; Stepanian et al., 2016; Ethier & Wilson, 2019). Notably, three‐dimensional hyperbolic localization of ultrasonic sounds is common and may be less challenging due to the smaller scale on which ultrasonic localization typically occurs (see Section “Placement”; e.g., Holderied & Helversen, 2003; Surlykke et al., 2009; Seibert et al., 2013). Third, direction‐of‐arrival methods can be used to produce a noise‐reduced recording of a target sound from a dense soundscape (see Section “Noise reduction”). Fourth, this noise reduction technique also enables automated detection of sounds in dense soundscapes. In contrast, hyperbolic methods require more human intervention during sound detection and noise reduction, especially for sound sources in dense soundscapes (but see Ethier & Wilson, 2019). Last, two open‐source, standalone programs for DOA estimation perform the entire pipeline of sound processing and position estimation, taking in recordings and putting out position estimates (Hedley et al., 2017; Suzuki et al., 2017). Although individual pieces of software for sound processing and position estimation are available for hyperbolic localization methods, there is currently no software available that performs the entire localization process.

These two methods are comparable in ease of placement for field deployment, coordinate localization performance in noisy soundscapes, accuracy of the position estimation algorithm, and potential for automation of the localization process. First, DOA arrays have the advantage over hyperbolic arrays of requiring a smaller number of ARUs to be deployed and measured (Section “Number of ARUs and microphones”). However, unlike in hyperbolic localization, a slight inaccuracy in measurement of the orientation of DOA arrays results in a large rotation of microphones, changing the DOA estimate (Girod, 2005; Trifa, 2006). Second, both methods are prone to errors in coordinate position estimation in noisy soundscapes. If two different sound sources are produced simultaneously, DOAs corresponding to the two different sources may be intersected, resulting in inaccurate localization (the data‐association problem; see Cobos et al., 2017). Hyperbolic localization in these conditions is prone to errors in calculation of time delays. In both cases, these problems can be mitigated to some extent by methods such as bandpassing the recording (e.g., Ali et al., 2009; Jones & Ratnam, 2009), manually removing overlapping sounds (e.g., Hedwig et al., 2018; Suzuki et al., 2017), and using automated algorithms to identify potentially inaccurate position estimations (e.g., Park & Kotun, 2018; Suzuki et al., 2018). Third, the accuracy of DOA methods is not well demonstrated, but at present it seems comparable to that of hyperbolic localization (Section “Position estimation”). Last, recent advances have been made in automating both localization methods (Ethier & Wilson, 2019; Wijers et al., 2019), but neither is truly automated yet.

Widespread adoption of acoustic localization, especially at large scales, is hindered by the requirement for time‐consuming human intervention in both sound detection and localization. Manual detection of sounds involves listening to recordings or looking through spectrograms of recordings to find sounds to be localized. Although the set of sounds to localize was detected automatically in many studies, even nominally automated detection methods often required human curation in practice. Curation methods included finding calls that were not detected by automated detectors (e.g., Hügel et al., 2017), and excluding detections that were false positives (e.g., Ali et al., 2007; Araya‐Salas et al., 2017), had poor signal‐to‐noise ratios (e.g., Mennill et al., 2012; Papin et al., 2018; Sumiya et al., 2017), or were overlapped by other vocalizations (e.g., Holderied, 2006; Krakauer et al., 2009). Designing an automated detector requires a priori knowledge of species acoustic properties and becomes more challenging as the number of species to be analyzed increases. Even after sound processing, many approaches involved manually checking, modifying, and removing problematic or inaccurate inputs to or outputs from localization algorithms, such as TDOAs, cross‐correlations between sounds, and DOA estimates (e.g., Ali et al., 2007; Campbell & Francis, 2012; McGregor et al., 1997; Spillmann et al., 2015; Surlykke & Kalko, 2008; Wahlberg et al., 2003; Wilson et al., 2014).

### 4.2 Next steps

In light of these findings, we suggest three developments to advance the field of acoustic localization: scalable recording hardware, open‐source localization software that performs well on noisy recordings, and animal sound classification.

First, we see a need for recording equipment that is widely available, inexpensive, self‐synchronizing, and low‐maintenance. None of the equipment used in the literature reviewed here meets all of these needs, although one recently developed system demonstrates many of these features (Wijers et al., 2019). The most common type of recording equipment used was the custom array, an array typically composed of individual microphones connected via cable for the purpose of the study. While these setups are less expensive to purchase than a dedicated wildlife recording system, running cables between microphones is time‐ and material‐intensive and disrupts the natural landscape. VoxNet, an academic array, had a self‐synchronizing capability, but was challenging to manufacture and resource‐intensive to deploy and maintain (Taylor et al., 2016). Wildlife recorders, such as those produced by Wildlife Acoustics, are commercially available with GPS synchronization and are built to be low‐maintenance, but their price of over 800USD may limit their availability. Less expensive, nonsynchronizing wildlife recorders exist, but have not yet been used in the localization literature (Darras et al., 2019). Lastly, while ARUs for DOA localization are commercially available due to broad applicability in other industries, they are typically not robust enough for use in wildlife settings. These arrays are intended for source separation of human conversation in indoor settings and lack the hardiness and low‐maintenance features that make wildlife recorders attractive. The multimicrophone construction of these ARUs consumes power and storage more quickly, requiring more frequent maintenance in the field. Furthermore, they lack waterproofing, and a slight change to the microphone orientation from wind or animal disturbance results in a large rotation of the microphones, changing the DOA estimate (Girod, 2005; Trifa, 2006). A potential path to a scalable recording platform is to combine the hardiness of wildlife recorders with the design of low‐cost recorders that are becoming more common on the market. For instance, ARUs such as the AudioMoth (Hill et al., 2017) and Raspberry Pi‐based devices (Segura‐Garcia et al., 2015) are available at only a fraction of the price of a typical Wildlife Acoustics recorder and can collect several hours of data daily for months before running out of storage space and battery. One very recently developed system, CARACAL, includes most of the desired features: It is open‐source, relatively inexpensive, and self‐synchronizing by GPS (Wijers et al., 2019). CARACAL's method intersected DOA estimates from 8 ARUs to estimate positions of gunshots and of three large mammal species. Each ARU included a planar arrangement of four microphones, a design which is applicable to estimation of long‐range detection of high‐amplitude sounds, and for short‐range detection of animals that show little vertical displacement, such as frogs. Due to the two‐dimensional design of the ARU, this system may not be suitable for animals that show large vertical displacement relative to array size, such as bats or birds (see Section “Number of ARUs and microphones”).

Second, we recommend three traits to prioritize in the development of localization software: robustness in noisy soundscapes, quantification of uncertainty in the localization pipeline, and open availability and usability for biologists. Progress toward automated acoustic localization has been hindered by the challenges of localizing sounds in noisy recordings. Hyperbolic methods typically require human intervention to reduce noise in audio or select relatively noise‐free portions of each recording. HARKBird, a MUSIC‐based method, has made promising progress toward achieving automation by automatically detecting sounds and separating overlapping sounds into multiple noise‐reduced recordings, but in practice these results are still manually reviewed. Advancements in noise reduction techniques may further improve the accuracy of localization results. While this software would ideally perform perfectly even in noisy scenarios, the widespread requirement for manual annotation hints at the difficulty of producing such a software. Thus, we suggest that software developers attempt to quantify uncertainty where it cannot be eliminated, including reporting uncertainty in sound detection or TDOA calculation, quantifying potential localization error (e.g., Sound Finder, Spiesberger, 2005; Wilson et al., 2014), and accounting for external factors such as reverberation (e.g., Gustafsson et al., 2003), source location relative to the center or boundaries of the array, and recorder positioning. Such a quantification of uncertainty allows practitioners to set thresholds for allowable certainty or prioritize sounds for manual review. Lastly, software must be widely available and easy for biologists to use. This means it should be well documented, include an intuitive graphical interface, and not require the use of expensive, specialized commercial applications such as MATLAB. Software should be open‐source such that it is able to be peer‐reviewed and freely modified by expert users. Notably, only 17 studies reported using open‐source or published localization software (Campbell & Francis, 2012; Hedley et al., 2017; Hedwig et al., 2018; Kershenbaum et al., 2019; Kojima et al., 2016, 2017; Matsubayashi et al., 2017; Papin et al. 2018; Park & Kotun, 2018; Rek, 2014; Spillmann et al., 2017; Suzuki et al., 2017, 2016, 2018; Wijers et al., 2019; Wilson et al., 2014; Wilson & Bayne, 2018).

Third, automated species classification via machine learning is necessary for truly automated animal localization. Most papers surveyed did not classify species automatically, and those that did attempt automated species classification usually classified only a single species (but see Vallejo & Taylor, 2009). However, some applications of localization, such as biodiversity monitoring, call for classifiers that can identify the dozens or more species likely to be present at each field site. Furthermore, current classifiers perform poorly at classifying species within a noisy soundscape, compared to classifiers only predicting clear “foreground” species in targeted audio recordings (Goeau, Kahl, Glotin, Planque, & Joly, 2018). Three potential methods for improving automated classification are source separation via beamforming (e.g., Jones & Ratnam, 2009; Kojima et al., 2017), machine learning to reduce background noise from single‐microphone recordings (e.g., Stoller, Ewert, & Dixon, 2018), and optimizing feature selection (e.g., careful selection of spectrogram parameters, Knight, Hernandez, Bayne, Bulitko, & Tucker, 2019). Another promising method to improve classification accuracy is using data augmentation to create artificial soundscapes, an approach that has successfully improved classification results in recent machine learning competitions (Goeau et al., 2018; Lasseck, 2018). If high accuracy cannot be achieved, classifiers with systematic and quantifiable error are preferred, as scientists can temper predictions by propagating uncertainty through the analysis (Kitzes & Schricker, 2019). In general, classification of animal sounds, especially bird sounds, has advanced significantly in recent years and remains an active field of research (reviewed in Priyadarshani, Marsland, et al., 2018).

In tandem with these suggestions, we reiterate the recommendation by Blumstein et al. (2011) to develop a common framework in which to share and compare automated bioacoustics methods. For acoustic localization in particular, such a framework could include improved availability of ground‐truth datasets for testing new sound processing and position estimation algorithms, and increased documentation of experiences, challenges, and pitfalls of particular methods. In Table 2, we suggest some features and results to report in future published applications of terrestrial wildlife localization. These include increased documentation of experiences, such as reporting effort information (e.g., Ethier & Wilson, 2019) and testing and reporting the performance of sound processing and position estimation methods (see Sections "Sound processing" and "Position estimation").

Automated acoustic localization has the potential to enable data collection at larger scales and with better accuracy than human observers, and can collect data that ARUs alone cannot capture. Data on individual locations can be used to study behavioral patterns of movement and migration, social interactions between individuals, fine scale habitat relationships, and overall species abundance and biodiversity. Furthermore, these methods are ripe for long‐term, large‐scale studies. They generate a permanent archival record of observations that can be reanalyzed in the future with updated algorithms, or reassessed to ask different questions. When aggregated over long time periods, location data can also be used to map territories and home range sizes, and could be used to estimate demographic rates based on territory occupation over time. Because of its flexibility, precision, and spatiotemporal scalability, automated acoustic localization may be an invaluable tool in studying many animals of conservation concern. Ultimately, ecology must embrace new data collection methods to address modern, large‐scale challenges of biodiversity loss and habitat change.

## CONFLICT OF INTEREST

The authors declare that there are no competing interests.

## AUTHOR CONTRIBUTION

**Tessa A. Rhinehart:** Conceptualization (lead); data curation (lead); formal analysis (lead); investigation (lead); methodology (lead); supervision (supporting); validation (supporting); visualization (lead); writing‐original draft (lead); and writing‐review & editing (lead). **Lauren M. Chronister:** Data curation (supporting); investigation (supporting); validation (supporting); and writing‐original draft (supporting). **Trieste Devlin:** Data curation (supporting); investigation (supporting); validation (supporting); and writing‐original draft (supporting). **Justin Kitzes:** Conceptualization (supporting); methodology (supporting); project administration (lead); resources (lead); supervision (lead); writing‐original draft (supporting); and writing‐review & editing (supporting). 

## Supporting information

Table S1‐S3

## Data Availability

The citation information for and analysis of all papers reviewed in this manuscript is provided in Table S1, which is available with the rest of our supplementary information at Figshare (doi.org/10.6084/m9.figshare.11944602).
